# Deep Reinforcement Learning for Collision-Aware Leader–Follower Formation Control

**DOI:** 10.3390/s26134162

**Published:** 2026-07-01

**Authors:** Sławomir Romaniuk, Jakub Budnik

**Affiliations:** Department of Automatic Control and Robotics, Faculty of Electrical Engineering, Bialystok University of Technology, 15-351 Bialystok, Poland; jakub.budnik2@gmail.com

**Keywords:** deep reinforcement learning, proximal policy optimization, leader–follower formation, collision avoidance, mobile robotics, MuJoCo, autonomous robots

## Abstract

This work tackles leader–follower navigation in scenarios where rapid, unpredictable leader maneuvers can trigger unsafe proximity or collisions. We apply Proximal Policy Optimization (PPO) to learn adaptive follower behavior capable of tracking the leader’s direction while considering a minimum-distance safety objective. A task-specific reward function—capturing formation constraints, collision avoidance, control-effort regularization, and responsiveness to sudden directional changes—enables effective policy learning. The results show that the PPO-based follower provides a favorable safety–tracking trade-off compared with the considered A2C and PID baselines under the simulated test conditions, although collision-free operation is not guaranteed in all episodes.

## 1. Introduction

Leader–follower formation control is a fundamental problem in cooperative mobile robotics, where a follower robot must maintain a prescribed relative configuration with respect to a moving leader. This paradigm is relevant to coordinated navigation, autonomous transportation, surveillance, warehouse robotics, and multi-robot exploration, where stable formation maintenance and safe inter-robot distances are essential [1,2]. In practice, abrupt changes in leader heading or velocity can increase tracking error and reduce the safety margin between robots.

Classical formation-control methods include trajectory-tracking and distance–bearing feedback control, PID-like controllers, LQR-based methods, consensus protocols, Lyapunov-based control, artificial potential fields, model predictive control, and control barrier function formulations [3,4,5,6,7,8,9]. They offer interpretability, computational efficiency, and, in many cases, formal stability or safety arguments. Recent leader–follower UAV studies also address collision avoidance and cooperative tracking under communication constraints [10,11]. However, compact local observations, nonlinear dynamics, abrupt maneuvers, and competing safety–tracking objectives can make the design of a single analytical controller difficult.

Reinforcement learning provides a data-driven framework for learning control policies through interaction with the environment [12,13]. Instead of relying solely on an explicitly derived control law, an RL agent optimizes a long-term objective defined by a reward function. Deep reinforcement learning has therefore gained increasing attention in robotic continuous-control, mapless-navigation, and collision-avoidance tasks, especially when the system is nonlinear, partially observable, or difficult to model precisely [14,15,16,17,18]. In multi-agent and swarm settings, reinforcement learning has also been investigated for decentralized coordination and collective behavior learning [19,20]. Among policy-gradient methods, Proximal Policy Optimization (PPO) is commonly used due to its stable policy updates and practical effectiveness in continuous-action problems [21]. Advantage Actor–Critic methods, such as A2C, are also frequently used as reference algorithms for evaluating policy-gradient approaches [22].

This paper investigates a compact-observation, safety-aware reinforcement learning framework for leader–follower formation control of a differential-drive mobile robot. The proposed framework is based on PPO, but the contribution of the work is not a modification of the PPO algorithm itself. Instead, the focus is placed on the design and evaluation of a modular reward function that combines formation tracking, collision avoidance, orientation alignment, and actuator-level control regularization. The follower receives a six-dimensional observation vector containing relative geometric and velocity-related information and produces two continuous wheel-control commands. The task is evaluated in a MuJoCo-based simulation environment [23], where the leader follows multiple trajectories containing both smooth and abrupt motion patterns. Although the proposed reward terms do not provide formal constraint-satisfaction guarantees, their safety-oriented design is motivated by the broader literature on safe and constrained reinforcement learning [24,25].

No physical equipment was used in this study; all experiments were conducted in simulation. The software version numbers were not available in the manuscript source. Therefore, the corresponding software websites and access dates are provided as requested by the journal: MuJoCo (https://mujoco.org, accessed on 28 June 2026), Gymnasium (https://gymnasium.farama.org, accessed on 28 June 2026), and Stable-Baselines3 (https://stable-baselines3.readthedocs.io, accessed on 28 June 2026).

The study is intended to clarify how individual reward components influence the behavior of the learned follower policy. For this purpose, several reward-weight variants are examined, allowing the relationship between tracking accuracy, safety, and control smoothness to be analyzed systematically. The evaluation considers not only average formation error but also safety violation rate, safety violation severity, collision count, velocity-tracking error, control smoothness, and post-maneuver recovery behavior. In addition, the learned PPO policy is compared with A2C and with a classical geometry-based leader–follower controller under the same observation, action, and trajectory conditions. The experimental analysis is further extended by robustness-oriented tests involving observation noise, actuation delay, and wheel-slip or action-scaling uncertainty. These tests are not intended to claim full sim-to-real validation, but rather to provide an initial assessment of the learned policy under more realistic disturbances, in line with the common observation that simulation-trained policies may be sensitive to the reality gap and to deployment-time uncertainty [26,27,28,29].

The main contributions of this work are as follows. The paper proposes a compact-observation reinforcement-learning framework for the collision-aware leader–follower formation control of a differential-drive robot under abrupt leader maneuvers. It also introduces a modular safety-aware reward formulation and evaluates it through a controlled reward-weight sensitivity study. The evaluation protocol combines formation accuracy, safety-violation rate and severity, velocity synchronization, control regularity, and post-maneuver recovery behavior, which makes it possible to analyze the learned policies beyond the accumulated reward alone. In addition, PPO is compared with A2C and with a classical distance–bearing PID controller under identical observation, action, and trajectory conditions. Finally, the study includes an observation-space ablation analysis and a test-time robustness evaluation under observation noise, actuation delay, and wheel-slip-like action scaling.

The remainder of this paper is organized as follows. Section 2 defines the leader–follower control problem, including the robot model, observation space, action space, and task assumptions. Section 3 presents the reinforcement learning methodology, reward formulation, neural-network architecture, and training configuration. Section 4 reports the experimental results, including reward-weight sensitivity analysis, baseline comparisons, generalization tests, and robustness evaluation. Finally, Section 5 concludes the paper and discusses limitations and directions for future work.

## 2. Problem Formulation

The considered task is a leader–follower formation-control problem in which a differential-drive mobile robot, hereafter referred to as the follower, must track a desired position behind a moving leader while maintaining a safe distance and producing feasible wheel-actuator commands. The task is formulated explicitly as a continuous-control decision-making problem with limited observations, rather than only as a descriptive simulation scenario. The formulation separates the full simulator state, the compact observation available to the policy, the continuous wheel-command action space, the leader-motion model, and the safety-oriented objective.

The control problem is represented as

(1) M=(S,O,A,P,h,r,γ,T),

where the following hold:

· S—full MuJoCo simulator state space;
· O—compact observation space available to the learning policy;
· A—continuous action space;
· $P(s_{t+1}|s_{t},a_{t})$—transition model induced by the MuJoCo physics engine and the imposed leader trajectory;
· h:S→O—observation function;
· $r(o_{t},a_{t})$—reward function;
· γ—discount factor;
· *T*—finite episode horizon.

At each time step *t*, the policy receives the observation $o_{t}=h\left(s_{t}\right)$, selects an action $a_{t}$, and the simulator advances the physical state according to the robot dynamics, contact interactions, actuator limits, and externally imposed leader motion. The goal is to learn a stochastic control policy

(2) $$\pi_{\theta }\left(a_{t}|o_{t}\right)$$

where the following hold:

· $\pi_{\theta }$—stochastic control policy;
· $a_{t}$—action selected at time step *t*;
· $o_{t}$—observation available at time step *t*;
· θ—policy-network parameters.

The policy is optimized by maximizing the expected discounted return

(3) $$J\left(\theta \right)=E_{\pi_{\theta }}\left[\sum_{t=0}^{T-1}\gamma^{t}r\left(o_{t},a_{t}\right)\right].$$

where the following hold:

· J(θ)—expected discounted return optimized by the policy;
· θ—policy-network parameters;
· $E_{\pi_{\theta }}\left[\cdot \right]$—expectation under policy $\pi_{\theta }$,
· γ—discount factor;
· $r(o_{t},a_{t})$—reward obtained from observation $o_{t}$ and action $a_{t}$.

**Figure 1:**
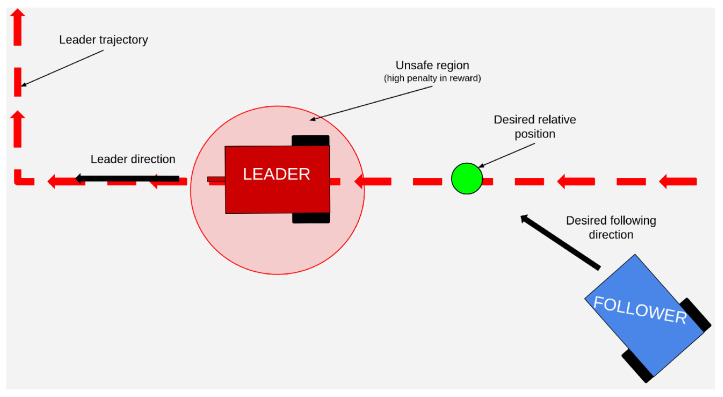
Leader–follower formation-control task. The follower is required to track the green reference point located behind the leader while avoiding the unsafe region around the leader.

The policy does not receive the complete MuJoCo state vector, global contact state, or all body poses and velocities. Instead, it operates on a six-dimensional vector of relative geometric and velocity-related quantities. Therefore, the implemented task can be interpreted as a compact-observation, partially observable leader–follower control problem. This compact representation is intentional: it reduces dependence on absolute world coordinates and forces the controller to react to the relative leader–follower configuration. The leader–follower task geometry is illustrated in Figure 1.

### 2.1. Simulated Objects and Physical Environment

The simulation environment was implemented in MuJoCo [23] and integrated with the Gymnasium interface [30]. The environment contains a differential-drive follower robot, a kinematic leader object, and an auxiliary formation reference point placed behind the leader. The follower is dynamically simulated, whereas the leader is controlled kinematically through a MuJoCo mocap body. This choice makes it possible to impose repeatable leader trajectories and to isolate the decision-making problem of the follower.

The MuJoCo model uses a fixed simulation time step of 0.01s. In the environment implementation, each control step consists of five internal MuJoCo integration substeps, resulting in an effective control interval of Δt=0.05s. Gravity is set to $9.81\;m/s^{2}$, and the ground is modeled as a horizontal plane with sliding, torsional, and rolling friction coefficients equal to 0.8, 0.01, and 0.001, respectively. This provides a controlled and repeatable physics-based testbed for evaluating the learned control policy.

The main geometric parameters of the simulated objects are summarized in Table 1. The dimensions are reported as full physical dimensions derived from the MuJoCo model.

### 2.2. Differential-Drive Follower Model

The follower is a differential-drive platform moving on a planar surface. Let

(4) $$q_{F}\left(t\right)={\left[x_{F}\left(t\right),y_{F}\left(t\right),\psi_{F}\left(t\right)\right]}^{⊤}$$

where the following hold:

· $q_{F}\left(t\right)$—planar pose of the follower;
· $x_{F}\left(t\right)$—longitudinal position of the follower body;
· $y_{F}\left(t\right)$—lateral position of the follower body;
· $\psi_{F}\left(t\right)$—yaw angle of the follower.

The ideal differential-drive kinematics are expressed as

(5) $${\dot{x}}_{F}=v_{F}\cos\psi_{F},\;{\dot{y}}_{F}=v_{F}\sin\psi_{F},\;{\dot{\psi }}_{F}=\omega_{F},$$

where the following hold:

· $v_{F}$—linear velocity of the follower;
· $\omega_{F}$—angular velocity of the follower;
· $\psi_{F}$—yaw angle of the follower.

These velocities are related to the left and right wheel angular velocities, $\omega_{L}$ and $\omega_{R}$, by 

(6) $$v_{F}=\frac{r_{w}}{2}\left(\omega_{R}+\omega_{L}\right),\;\omega_{F}=\frac{r_{w}}{L}\left(\omega_{R}-\omega_{L}\right),$$

where the following hold:

· $r_{w}$—wheel radius, equal to 0.07m;
· *L*—lateral distance between the two drive wheels, approximately 0.346m;
· $\omega_{L}$—angular velocity of the left wheel;
· $\omega_{R}$—angular velocity of the right wheel.

The above kinematic equations clarify the relationship between wheel commands and planar motion. In the actual implementation, however, the follower motion is generated by MuJoCo through numerical integration of the full physical model, including body inertia, gravity, wheel–ground contact, friction, joint damping, and actuator constraints. The policy therefore controls the robot through wheel actuators, while the resulting motion is determined by the simulator dynamics rather than by a purely kinematic update.

### 2.3. Leader Motion and Formation Reference

The leader position in the horizontal plane is denoted by

(7) $$p_{L}\left(t\right)={\left[x_{L}\left(t\right),y_{L}\left(t\right)\right]}^{⊤}.$$

where the following hold:

· $p_{L}\left(t\right)$—planar leader position at time step *t*;
· $x_{L}\left(t\right)$—longitudinal leader position;
· $y_{L}\left(t\right)$—lateral leader position.

During training, the leader follows a set of predefined but diverse trajectories. These trajectories include circular, elliptical, sinusoidal, spiral, square, polygonal, and Lissajous-like paths. They are designed to expose the follower to both smooth and abrupt leader maneuvers, including changes in curvature, heading, and speed. The leader is not dynamically affected by the follower, and no direct communication between the leader and the follower is assumed.

The instantaneous direction of leader motion is estimated from consecutive leader positions:

(8) $${\hat{d}}_{L}\left(t\right)=\frac{p_{L}\left(t\right)-p_{L}\left(t-\Delta t\right)}{∥p_{L}\left(t\right)-p_{L}{\left(t-\Delta t\right)∥}_{2}},$$

where the following hold:

· ${\hat{d}}_{L}\left(t\right)$—unit vector of the instantaneous leader-motion direction,
· $p_{L}\left(t\right)$—current leader position,
· $p_{L}\left(t-\Delta t\right)$—leader position at the previous control step,
· Δt—control interval,
· ${∥\cdot ∥}_{2}$—Euclidean norm.

This expression is used when the leader displacement is nonzero. The desired formation point is located behind the leader along the opposite direction of motion:

(9) $$p_{\mathrm{ref}}\left(t\right)=p_{L}\left(t\right)-d_{\mathrm{ref}}{\hat{d}}_{L}\left(t\right),$$

where the following hold:

· $p_{\mathrm{ref}}\left(t\right)$—desired reference position behind the leader;
· $p_{L}\left(t\right)$—leader position in the horizontal plane;
· $d_{\mathrm{ref}}$—desired behind-leader offset, equal to 1.0m;
· ${\hat{d}}_{L}\left(t\right)$—unit vector of the instantaneous leader-motion direction.

The corresponding formation error is

(10) $$e_{\mathrm{form}}\left(t\right)={∥p_{F}\left(t\right)-p_{\mathrm{ref}}\left(t\right)∥}_{2},$$

where the following hold:

· $e_{\mathrm{form}}\left(t\right)$—instantaneous formation error;
· $p_{F}\left(t\right)$—planar follower position;
· $p_{\mathrm{ref}}\left(t\right)$—desired reference position behind the leader;
· ${∥\cdot ∥}_{2}$—Euclidean norm.

Safety is defined with respect to the physical distance between the follower and the leader:

(11) $$d_{L}\left(t\right)={∥p_{F}\left(t\right)-p_{L}\left(t\right)∥}_{2}.$$

The follower is considered to violate the safety constraint when

(12) $$d_{L}\left(t\right)<d_{\mathrm{safe}},$$

where the following hold:

· $d_{L}\left(t\right)$—direct leader–follower distance;
· $d_{\mathrm{safe}}$—minimum admissible leader–follower distance, equal to 0.5m in the nominal environment configuration.

The learning objective therefore combines formation tracking with avoidance of excessive proximity to the leader.

### 2.4. Random-Seed Training Stability

To assess the repeatability of the learning process, an additional random-seed training stability test was performed. The purpose of this experiment was not to replace the full nominal benchmark, but to verify whether the learning trend observed for the selected PPO configuration is preserved under different random initializations. The test was conducted for the safety-oriented PPO_R3 variant, which was previously identified as one of the representative reward configurations.

Three independent shortened PPO training runs were performed using random seeds

(13) s∈{0,1,2}.

where the following hold:

· *s*—base random seed used for a given training run.

In each run, the reward weights, policy architecture, observation vector, action space, PPO hyperparameters, and training trajectories were kept unchanged. Only the random seed was modified. The seed affects the initial policy parameters, stochastic action sampling during training, minibatch ordering during PPO optimization, and randomized initial follower poses at environment reset. For parallel training environments, the environment-specific seed is shifted by the environment index:

(14) $$s_{i}=s+i,$$

where the following hold:

· $s_{i}$—seed assigned to the *i*-th parallel environment;
· *s*—base random seed used for the training run;
· *i*—index of the parallel environment.

Because this experiment was intended as a supplementary repeatability check, a shortened training horizon was used. Each run was trained for approximately $5.08\times 10^{5}$ environment steps, corresponding to 336 logged training episodes. Therefore, the results should be interpreted as evidence of qualitative training repeatability rather than as a full statistical validation of final policy performance.

The training reward is negative because the reward function is dominated by penalty terms. Thus, values closer to zero indicate a lower accumulated weighted penalty and better optimization of the corresponding training objective. For each seed, the initial reward was computed as the mean reward over the first 50 logged episodes, whereas the final reward was computed as the mean reward over the last 50 logged episodes. The final formation error, velocity-tracking error, and smoothness were computed from the last 10% of logged training-metric samples.

Table 2 shows that all three shortened PPO_R3 training runs improved the training objective. The mean final reward increased from −12,421.2 to −6829.1, corresponding to an average relative improvement of 44.4%±6.7%. Since all three runs show a positive reward improvement, the result suggests that the observed learning trend is not limited to a single favorable random seed.

The final reward still exhibits noticeable variation across seeds. The best final reward was obtained for seed 0, whereas seed 2 resulted in a lower final reward and a higher final formation error. This indicates that the shortened training process remains sensitive to stochastic initialization and sampling. Therefore, the experiment should not be interpreted as proof of full quantitative convergence across seeds.

The formation-error results show moderate variability. Seeds 0 and 1 resulted in almost identical final formation errors, equal to 1.727m and 1.728m, respectively. Seed 2 produced a higher final error of 2.003m. The across-seed mean formation error was 1.819±0.159m. This suggests that the training process produces qualitatively similar, although not identical, formation-tracking behavior under the considered shortened training horizon.

The velocity-tracking error and smoothness metrics are more consistent across seeds. The velocity-tracking error remains in the range from 1.269m/s to 1.383m/s, while the smoothness metric remains between 1.600 and 1.668. This indicates that the actuator-level behavior is relatively repeatable in the shortened training runs. However, the velocity-tracking error remains higher than in the fully trained nominal models, which confirms that the shortened random-seed experiment should be treated as a repeatability check rather than as a final performance benchmark.

Overall, the random-seed training experiment indicates that PPO_R3 exhibits a repeatable qualitative learning trend across the tested seeds s∈{0,1,2}. All runs improved the reward objective, and the velocity-related metrics remained relatively consistent. At the same time, the differences in final reward and formation error show that shortened training still introduces moderate variability. Consequently, this experiment supports the reproducibility of the learning process at the trend level, while the main task-level conclusions should remain based on the fully trained policies evaluated in the nominal comparison.

### 2.5. Observation Space

The policy receives a compact six-dimensional observation vector

(15) $$o_{t}={\left[\alpha_{t},{\bar{d}}_{L,t},{\bar{\omega }}_{F,t},{\bar{v}}_{F,t},{\bar{v}}_{L,t},\Delta \psi_{t}\right]}^{⊤}\in O\subset R^{6}.$$

where the following hold:

· $o_{t}$—compact observation vector at time step *t*;
· $\alpha_{t}$—relative bearing angle from the follower heading to the leader position;
· ${\bar{d}}_{L,t}$—normalized follower–leader distance;
· ${\bar{\omega }}_{F,t}$—normalized yaw rate of the follower;
· ${\bar{v}}_{F,t}$—normalized forward velocity of the follower;
· ${\bar{v}}_{L,t}$—normalized leader speed;
· $\Delta \psi_{t}$—relative direction of leader motion with respect to the follower heading.

The components correspond directly to the quantities returned by the environment implementation. The normalized follower–leader distance is

(16) $${\bar{d}}_{L,t}=\mathrm{clip}\left(\frac{d_{L}\left(t\right)}{d_{\max}},0,1\right),$$

where the following hold:

· $d_{L}\left(t\right)$—Euclidean leader–follower distance;
· $d_{\max}$—maximum distance used for normalization, equal to 5.0m;
· clip(·)—clipping operator limiting the value to the stated interval.

The normalized yaw rate of the follower is

(17) $${\bar{\omega }}_{F,t}=\mathrm{clip}\left(\frac{\omega_{F}\left(t\right)}{\omega_{\max}},-1,1\right),$$

where the following hold:

· $\omega_{F}\left(t\right)$—yaw rate of the follower;
· $\omega_{\max}$—maximum yaw-rate magnitude used for normalization, equal to 5.0rad/s.

The normalized forward velocity of the follower is

(18) $${\bar{v}}_{F,t}=\mathrm{clip}\left(\frac{v_{F}\left(t\right)}{v_{F,\max}},-1,1\right),$$

where the following hold:

· $v_{F}\left(t\right)$—forward velocity of the follower;
· $v_{F,\max}$—maximum follower velocity used for normalization, equal to 3.0m/s.

The normalized leader speed is

(19) $${\bar{v}}_{L,t}=\mathrm{clip}\left(\frac{v_{L}\left(t\right)}{v_{L,\max}},0,1\right),$$

where the following hold:

· $v_{L}\left(t\right)$—leader speed;
· $v_{L,\max}$—maximum leader speed used for normalization, equal to 3.0m/s.

The relative direction of leader motion with respect to the follower heading is

(20) $$\Delta \psi_{t}=\mathrm{atan}2\left(\sin\left(\psi_{L}\left(t\right)-\psi_{F}\left(t\right)\right),\cos\left(\psi_{L}\left(t\right)-\psi_{F}\left(t\right)\right)\right),$$

where the following hold:

· $\Delta \psi_{t}$—wrapped relative direction of leader motion with respect to the follower heading;
· $\psi_{L}\left(t\right)$—direction of leader motion;
· $\psi_{F}\left(t\right)$—follower yaw angle.

The resulting observation space is bounded by

(21) O=[−π,π]×[0,1]×[−1,1]×[−1,1]×[0,1]×[−π,π].

This observation vector intentionally excludes absolute world coordinates and full simulator state variables. As a result, the learned policy must infer suitable wheel commands from relative leader–follower geometry and velocity information only.

### 2.6. Action Space and Actuator Mapping

The action selected by the policy is a two-dimensional continuous vector

(22) $$a_{t}={\left[a_{L,t},a_{R,t}\right]}^{⊤},$$

where the following hold:

· $a_{t}$—continuous two-dimensional action vector;
· $a_{L,t}$—normalized command for the left wheel actuator;
· $a_{R,t}$—normalized command for the right wheel actuator.

The action space is

(23) $$A={\left[-1,1\right]}^{2}.$$

Before being applied to the MuJoCo actuators, the normalized action is clipped to [−1,1] and linearly mapped to the actuator-control range:

(24) $$u_{i,t}^{\mathrm{phys}}=u_{i}^{\min}+\frac{a_{i,t}+1}{2}\left(u_{i}^{\max}-u_{i}^{\min}\right),\;i\in \left\{L,R\right\}.$$

where the following hold:

· $u_{i,t}^{\mathrm{phys}}$—physical actuator command applied to wheel *i*;
· $a_{i,t}$—normalized policy output for wheel *i*;
· $u_{i}^{\min}$—lower actuator-control limit for wheel *i*;
· $u_{i}^{\max}$—upper actuator-control limit for wheel *i*;
· i∈{L,R}—index denoting the left or right wheel actuator.

For the implemented robot model, the actuator limits are

(25) $$u_{L}^{\min}=u_{R}^{\min}=-10,\;u_{L}^{\max}=u_{R}^{\max}=10.$$

where the following hold:

· $u_{L}^{\min}$—lower control limit of the left wheel actuator;
· $u_{R}^{\min}$—lower control limit of the right wheel actuator;
· $u_{L}^{\max}$—upper control limit of the left wheel actuator;
· $u_{R}^{\max}$—upper control limit of the right wheel actuator.

This explicit mapping ensures that the policy output remains normalized, while the simulator receives physically bounded actuator commands.

### 2.7. Transition Dynamics

The transition model $P(s_{t+1}|s_{t},a_{t})$ is not specified analytically, because the physical evolution of the system is computed by the MuJoCo simulator. Let $s_{t}$ denote the full simulator state, including body poses, velocities, joint states, contact states, and internal MuJoCo variables. The transition can be written compactly as

(26) $$s_{t+1}=f_{\mathrm{MJ}}\left(s_{t},u_{t}^{\mathrm{phys}},p_{L}\left(t\right)\right),$$

where the following hold:

· $s_{t}$—full simulator state at time step *t*;
· $u_{t}^{\mathrm{phys}}$—physical actuator command vector applied to the follower;
· $p_{L}\left(t\right)$—externally imposed leader position;
· $f_{\mathrm{MJ}}\left(\cdot \right)$—numerical MuJoCo integration procedure.

The observation used by the policy is then obtained through

(27) $$o_{t}=h\left(s_{t},p_{L}\left(t\right)\right).$$

where the following hold:

· $o_{t}$—compact observation vector available to the policy;
· h(·)—observation function;
· $s_{t}$—full simulator state;
· $p_{L}\left(t\right)$—leader position.

This distinction is important because the simulator internally evolves a high-dimensional physical state, whereas the policy only observes the compact vector defined above.

### 2.8. Observation and Actuation Uncertainty

The nominal training environment uses direct observations and direct execution of wheel commands. However, to define the robustness evaluation considered in the revised experimental protocol, observation and actuation uncertainties can be introduced at test time. A noisy observation can be expressed as

(28) $${\tilde{o}}_{t}=o_{t}+\epsilon_{t},$$

where the following hold:

· ${\tilde{o}}_{t}$—noisy observation vector;
· $o_{t}$—nominal observation vector;
· $\epsilon_{t}$—observation-noise vector.

In particular, distance and bearing measurements can be perturbed as

(29) $${\tilde{d}}_{L}\left(t\right)=d_{L}\left(t\right)+\epsilon_{d},\;\epsilon_{d}∼N\left(0,\sigma_{d}^{2}\right),$$

(30) $${\tilde{\alpha }}_{t}=\alpha_{t}+\epsilon_{\alpha },\;\epsilon_{\alpha }∼N\left(0,\sigma_{\alpha }^{2}\right).$$

where the following hold:

· ${\tilde{d}}_{L}\left(t\right)$—noisy leader–follower distance;
· $d_{L}\left(t\right)$—nominal leader–follower distance;
· $\epsilon_{d}$—distance-measurement noise;
· $\sigma_{d}$—standard deviation of distance noise;
· ${\tilde{\alpha }}_{t}$—noisy relative bearing angle;
· $\alpha_{t}$—nominal relative bearing angle;
· $\epsilon_{\alpha }$—bearing-measurement noise;
· $\sigma_{\alpha }$—standard deviation of bearing noise.

Actuator delay can be modeled by executing an earlier action instead of the currently selected command:

(31) $$a_{t}^{\mathrm{exec}}=a_{t-k},$$

where the following hold:

· $a_{t}^{\mathrm{exec}}$—action actually executed by the simulator at time step *t*;
· $a_{t-k}$—action generated *k* control steps earlier;
· *k*—delay in control steps.

Wheel-slip-like actuation uncertainty can be approximated by action scaling:

(32) $$a_{t}^{\mathrm{exec}}=\lambda_{t}a_{t},\;\lambda_{t}∼U\left(\lambda_{\min},\lambda_{\max}\right).$$

where the following hold:

· $a_{t}^{\mathrm{exec}}$—scaled action executed by the simulator;
· $a_{t}$—nominal action generated by the policy;
· $\lambda_{t}$—random action-scaling coefficient;
· $U(\lambda_{\min},\lambda_{\max})$—uniform distribution over the scaling interval.

These perturbation models are not part of the nominal training setting, but they formalize the noise, delay, and actuator-effectiveness disturbances used to evaluate robustness of the learned policy.

### 2.9. Task Assumptions

The formulation assumes that the follower moves on a planar surface and is controlled only through two wheel actuators. The leader motion is imposed by the simulator and is not dynamically affected by the follower, which makes the leader trajectory repeatable across training and evaluation runs. No direct communication between the leader and the follower is available. Consequently, the policy receives only the compact six-dimensional observation vector defined above and does not have access to the full MuJoCo simulator state, global body poses, or contact information.

The desired formation point is located behind the leader along the direction opposite to its instantaneous motion. Safety is defined by a minimum admissible leader–follower distance $d_{\mathrm{safe}}$. The nominal training process is conducted in a controlled MuJoCo environment, whereas the robustness evaluation introduces additional observation noise, action delay, and action-scaling uncertainty. These assumptions make the task sufficiently constrained for repeatable simulation-based learning while still preserving the central difficulty of compact-observation leader–follower control under changing leader maneuvers.

This formulation provides a rigorous and reproducible basis for the proposed safety-aware reinforcement-learning controller. It also explicitly identifies the state hidden inside the simulator, the limited observation available to the policy, the continuous wheel-command interface, the leader-motion assumptions, and the safety constraint that governs the leader–follower interaction.

## 3. Methodology

This section describes the reinforcement-learning methodology used to train the follower robot in the leader–follower formation-control task. The environment, observation space, action space, and transition model were defined in Section 2. Therefore, the present section focuses on the learning architecture, action-to-actuator mapping, reward formulation, training setup, and evaluation metrics. The main controller was trained using Proximal Policy Optimization (PPO), while Advantage Actor–Critic (A2C) was used as a reference actor–critic algorithm in the comparative experiments [21,22].

### 3.1. Policy Architecture

The control policy receives a compact six-dimensional observation vector and returns two continuous wheel-actuator commands. The observation vector used by the neural network is defined as

(33) $$o_{t}={\left[\alpha_{t},{\bar{d}}_{L,t},{\bar{\omega }}_{F,t},{\bar{v}}_{F,t},{\bar{v}}_{L,t},\Delta \psi_{t}\right]}^{⊤}\in R^{6}.$$

where the following hold:

· $o_{t}$—compact observation vector at time step *t*;
· $\alpha_{t}$—relative angle from the follower heading to the leader;
· ${\bar{d}}_{L,t}$—normalized leader–follower distance;
· ${\bar{\omega }}_{F,t}$—normalized yaw rate of the follower;
· ${\bar{v}}_{F,t}$—normalized forward velocity of the follower;
· ${\bar{v}}_{L,t}$—normalized leader speed;
· $\Delta \psi_{t}$—relative direction of leader motion with respect to the follower heading.

The angular components are provided directly in radians within [−π,π]. The remaining scalar quantities are clipped and normalized as follows:

(34) $${\bar{d}}_{L,t}=\mathrm{clip}\left(\frac{d_{L}\left(t\right)}{d_{\max}},0,1\right),\;d_{\max}=5.0\;m.$$

(35) $${\bar{\omega }}_{F,t}=\mathrm{clip}\left(\frac{\omega_{F}\left(t\right)}{\omega_{\max}},-1,1\right),\;\omega_{\max}=5.0\;\mathrm{rad}/s.$$

(36) $${\bar{v}}_{F,t}=\mathrm{clip}\left(\frac{v_{F}\left(t\right)}{v_{F,\max}},-1,1\right),\;v_{F,\max}=3.0\;m/s.$$

(37) $${\bar{v}}_{L,t}=\mathrm{clip}\left(\frac{v_{L}\left(t\right)}{v_{L,\max}},0,1\right),\;v_{L,\max}=3.0\;m/s.$$

where the following hold:

· ${\bar{d}}_{L,t}$—normalized leader–follower distance;
· $d_{L}\left(t\right)$—Euclidean leader–follower distance;
· $d_{\max}$—maximum distance used for normalization;
· ${\bar{\omega }}_{F,t}$—normalized yaw rate of the follower;
· $\omega_{F}\left(t\right)$—yaw rate of the follower;
· $\omega_{\max}$—maximum yaw-rate magnitude used for normalization;
· ${\bar{v}}_{F,t}$—normalized forward velocity of the follower;
· $v_{F}\left(t\right)$—forward velocity of the follower;
· $v_{F,\max}$—maximum follower velocity used for normalization;
· ${\bar{v}}_{L,t}$—normalized leader speed;
· $v_{L}\left(t\right)$—leader speed;
· $v_{L,\max}$—maximum leader speed used for normalization;
· clip(·)—clipping operator limiting each value to the stated interval.

The relative direction of leader motion is computed using a wrapped angular difference:

(38) $$\Delta \psi_{t}=\mathrm{atan2}\left(\sin\left(\psi_{L}\left(t\right)-\psi_{F}\left(t\right)\right),\cos\left(\psi_{L}\left(t\right)-\psi_{F}\left(t\right)\right)\right).$$

where the following hold:

· $\Delta \psi_{t}$—wrapped relative direction of leader motion with respect to the follower heading;
· $\psi_{L}\left(t\right)$—instantaneous direction of leader motion;
· $\psi_{F}\left(t\right)$—follower yaw angle;
· atan2(·)—four-quadrant arctangent used to keep the angular difference wrapped.

The policy output is a two-dimensional continuous action vector

(39) $$a_{t}={\left[a_{L,t},a_{R,t}\right]}^{⊤}\in {\left[-1,1\right]}^{2},$$

where the following hold:

· $a_{t}$—continuous two-dimensional action vector;
· $a_{L,t}$—normalized left-wheel command before actuator scaling;
· $a_{R,t}$—normalized right-wheel command before actuator scaling.

The controller was implemented using the MlpPolicy architecture from Stable-Baselines3 [31]. Since no custom policy architecture was specified, the default feed-forward actor–critic multilayer perceptron was used. The actor and critic use separate multilayer perceptrons with two hidden layers of 64 units each and hyperbolic tangent activation functions. The actor maps the six-dimensional observation vector to the parameters of a diagonal Gaussian continuous action distribution, whereas the critic estimates the scalar value function. The log standard deviation of the action distribution is learned together with the policy parameters. No recurrent layers, memory modules, or image encoders were used. Table 3 summarizes the neural-network input, output, and policy representation.

### 3.2. Action-to-Actuator Mapping

The neural network does not directly output physical wheel torques or angular velocities. Instead, it generates normalized actions that are clipped to [−1,1] and then linearly mapped to the actuator control range defined in the MuJoCo model. This mapping is implemented as

(40) $$u_{i,t}^{\mathrm{phys}}=u_{i}^{\min}+\frac{a_{i,t}+1}{2}\left(u_{i}^{\max}-u_{i}^{\min}\right),\;i\in \left\{L,R\right\}.$$

where the following hold:

· $u_{i,t}^{\mathrm{phys}}$—physical actuator command applied to wheel *i*;
· $a_{i,t}$—normalized policy output for wheel *i*;
· $u_{i}^{\min}$—lower actuator-control limit for wheel *i*;
· $u_{i}^{\max}$—upper actuator-control limit for wheel *i*;
· i∈{L,R}—index denoting the left or right wheel actuator.

For the simulated robot, both wheel actuators use the same control interval:

(41) $$u_{L}^{\min}=u_{R}^{\min}=-10,\;u_{L}^{\max}=u_{R}^{\max}=10.$$

where the following hold:

· $u_{L}^{\min}$—lower control limit of the left wheel actuator;
· $u_{R}^{\min}$—lower control limit of the right wheel actuator;
· $u_{L}^{\max}$—upper control limit of the left wheel actuator;
· $u_{R}^{\max}$—upper control limit of the right wheel actuator.

Thus, for the present actuator range, the mapping is equivalent to $u_{i,t}^{\mathrm{phys}}=10a_{i,t}$. This formulation keeps the reinforcement-learning action space normalized while ensuring that all commands applied to the MuJoCo model remain within the physical actuator limits.

### 3.3. Policy Optimization

PPO was selected as the main learning algorithm because it limits excessively large policy updates through a clipped surrogate objective. This property is useful in the considered task, where unstable updates may lead to abrupt changes in wheel commands and unsafe behavior near the leader. The PPO objective is written as

(42) $$L^{\mathrm{CLIP}}\left(\theta \right)=E_{t}\left[\min\left(\rho_{t}\left(\theta \right){\hat{A}}_{t},\mathrm{clip}\left(\rho_{t}\left(\theta \right),1-\epsilon ,1+\epsilon \right){\hat{A}}_{t}\right)\right].$$

where the following hold:

· $L^{\mathrm{CLIP}}\left(\theta \right)$—clipped PPO surrogate objective;
· θ—parameters of the policy network;
· $\rho_{t}\left(\theta \right)$—probability ratio between the updated and previous policies;
· ${\hat{A}}_{t}$—estimated advantage at time step *t*;
· ϵ—clipping parameter limiting the policy update;
· $E_{t}\left[\cdot \right]$—empirical expectation over sampled rollout transitions.

The probability ratio used in the PPO objective is defined as

(43) $$\rho_{t}\left(\theta \right)=\frac{\pi_{\theta }\left(a_{t}|o_{t}\right)}{\pi_{\theta_{\mathrm{old}}}\left(a_{t}|o_{t}\right)}.$$

where the following hold:

· $\rho_{t}\left(\theta \right)$—policy probability ratio at time step *t*;
· $\pi_{\theta }\left(a_{t}|o_{t}\right)$—probability of action $a_{t}$ under the updated policy;
· $\pi_{\theta_{\mathrm{old}}}\left(a_{t}|o_{t}\right)$—probability of action $a_{t}$ under the previous policy;
· $o_{t}$—observation available to the policy;
· $a_{t}$—action selected by the policy.

A2C was used only as a reference actor–critic method. Its actor update is based on the advantage estimate

(44) $$A_{t}=R_{t}-V_{\phi }\left(o_{t}\right).$$

where the following hold:

· $A_{t}$—advantage estimate at time step *t*;
· $R_{t}$—discounted return;
· $V_{\phi }\left(o_{t}\right)$—critic estimate of the value function;
· ϕ—parameters of the critic network.

In contrast to PPO, A2C does not constrain the policy update using a clipped probability ratio. This makes it a useful baseline for assessing whether the more conservative update mechanism of PPO improves training stability and safety in the considered leader–follower task.

### 3.4. Classical Distance–Bearing PID Baseline

The classical baseline used in this study is a distance–bearing PID controller, following the general feedback-control principle of PID regulation and classical tracking control for mobile robots [8,9]. It represents a closed-loop, error-feedback type of leader–follower tracking: at every control step, the follower computes its position error with respect to the moving reference point located behind the leader and then generates wheel commands intended to reduce this error. In contrast to the learned PPO and A2C policies, the PID controller does not optimize a reward function, does not adapt its gains during evaluation, and does not predict future leader motion. Its behavior is therefore reactive and is determined by the current and past tracking errors together with fixed controller gains.

The basic PID principle combines three terms. The proportional term reacts to the current error, the integral term accumulates past error and can reduce steady-state offset, and the derivative term reacts to the rate of error change, which can damp oscillatory responses. In the present distance–bearing formulation, two error signals are used: the distance error to the desired behind-leader point and the bearing error between the follower heading and the direction toward that point. Let

(45) $$e_{d}\left(t\right)={∥p_{\mathrm{ref}}\left(t\right)-p_{F}\left(t\right)∥}_{2},\;e_{\alpha }\left(t\right)=\mathrm{atan2}\left(\sin\left(\alpha \left(t\right)-\psi_{F}\left(t\right)\right),\cos\left(\alpha \left(t\right)-\psi_{F}\left(t\right)\right)\right),$$

where $\alpha \left(t\right)=\mathrm{atan2}(y_{\mathrm{ref}}\left(t\right)-y_{F}\left(t\right),x_{\mathrm{ref}}\left(t\right)-x_{F}\left(t\right))$ is the direction from the follower to the desired reference point. The linear and angular commands are then computed as

(46) $$v_{\mathrm{PID}}\left(t\right)=K_{P,d}e_{d}\left(t\right)+K_{I,d}\sum_{\tau =0}^{t}e_{d}\left(\tau \right)\Delta t+K_{D,d}\frac{e_{d}\left(t\right)-e_{d}\left(t-1\right)}{\Delta t},$$

(47) $$\omega_{\mathrm{PID}}\left(t\right)=K_{P,\alpha }e_{\alpha }\left(t\right)+K_{I,\alpha }\sum_{\tau =0}^{t}e_{\alpha }\left(\tau \right)\Delta t+K_{D,\alpha }\frac{e_{\alpha }\left(t\right)-e_{\alpha }\left(t-1\right)}{\Delta t}.$$

The commanded linear and angular velocities are converted to left and right differential-drive wheel commands using the standard inverse kinematic relation and are clipped to the same actuator limits as the learning-based controllers. In qualitative terms, this controller performs geometric point following: it attempts to drive the follower toward the instantaneous reference point behind the leader while aligning the follower with that direction. This makes it a useful interpretable non-learning baseline, but it also means that the controller is sensitive to gain tuning and may lag behind abrupt leader maneuvers because it responds only after the distance and bearing errors have appeared.

### 3.5. Reward Function

The reward function follows the implementation used in the environment and combines formation tracking, safety, control-effort regularization, and bearing-angle shaping. The reference point is located behind the leader along the opposite direction of leader motion:

(48) $$p_{\mathrm{ref}}\left(t\right)=p_{L}\left(t\right)-b\;{\hat{d}}_{L}\left(t\right),\;b=1.0\;m.$$

where the following hold:

· $p_{\mathrm{ref}}\left(t\right)$—desired reference position behind the leader;
· $p_{L}\left(t\right)$—leader position at time step *t*;
· *b*—desired behind-leader offset;
· ${\hat{d}}_{L}\left(t\right)$—unit vector representing the instantaneous direction of leader motion.

The distance to this point is

(49) $$d_{\mathrm{behind}}\left(t\right)={∥p_{F}\left(t\right)-p_{\mathrm{ref}}\left(t\right)∥}_{2}.$$

where the following hold:

· $d_{\mathrm{behind}}\left(t\right)$—distance between the follower and the reference point behind the leader;
· $p_{F}\left(t\right)$—follower position at time step *t*;
· $p_{\mathrm{ref}}\left(t\right)$—desired reference position behind the leader;
· ${∥\cdot ∥}_{2}$—Euclidean norm.

The distance used for safety evaluation is the direct leader–follower distance:

(50) $$d_{L}\left(t\right)={∥p_{F}\left(t\right)-p_{L}\left(t\right)∥}_{2}.$$

where the following hold:

· $d_{L}\left(t\right)$—direct distance between the follower and the leader;
· $p_{F}\left(t\right)$—follower position at time step *t*;
· $p_{L}\left(t\right)$—leader position at time step *t*;
· ${∥\cdot ∥}_{2}$—Euclidean norm.

The reward at time step *t* is defined as

(51) $$r_{t}=-w_{f}d_{\mathrm{behind}}\left(t\right)-w_{c}\max\left(0,d_{\mathrm{safe}}-d_{L}\left(t\right)\right)-w_{a}\sum_{i\in \{L,R\}}a_{i,t}^{2}-w_{\theta }\left|\alpha_{t}\right|.$$

where the following hold:

· $r_{t}$—reward value at time step *t*;
· $w_{f}$—weight of the formation-tracking penalty;
· $d_{\mathrm{behind}}\left(t\right)$—distance from the follower to the desired reference point behind the leader;
· $w_{c}$—weight of the safety penalty;
· $d_{\mathrm{safe}}$—minimum admissible leader–follower distance;
· $d_{L}\left(t\right)$—direct leader–follower distance;
· $w_{a}$—weight of the control-effort regularization penalty;
· $a_{i,t}$—normalized action applied to wheel actuator *i*;
· $w_{\theta }$—weight of the bearing-angle shaping term;
· $\alpha_{t}$—relative bearing angle from the follower heading to the leader.

The first term penalizes deviation from the desired position behind the leader. The second term is activated only when the follower enters the unsafe region around the leader. The third term discourages excessive wheel-command magnitudes and should therefore be interpreted as control-effort regularization rather than a direct smoothness penalty. The final term provides an additional bearing-angle shaping signal. The nominal safety threshold is

(52) $$d_{\mathrm{safe}}=0.5\;m.$$

where the following hold:

· $d_{\mathrm{safe}}$—minimum admissible distance between the follower and the leader.

The reward variants R1–R7 are treated as a controlled reward-weight sensitivity study. In this interpretation, the variants are not independent manual tunings, but systematic modifications of individual reward components. R1 is the baseline configuration, R2 and R3 increase the safety penalty, R4 increases the formation-tracking weight, R5 and R6 modify the control-effort regularization term, and R7 increases the bearing-angle shaping weight. The corresponding reward-weight variants are listed in Table 4.

### 3.6. Training Setup

Training was performed in sixteen parallel MuJoCo environments. Each environment used a different leader trajectory, with path identifiers from 0 to 15. This setup exposes the policy to circular, elliptical, sinusoidal, spiral, polygonal, figure-eight, and Lissajous-like leader motions. The leader trajectory is imposed by the simulation, while the follower is controlled only through the learned wheel-command policy.

Each PPO model was trained for $3\times 10^{6}$ environment steps. The main training parameters are summarized in Table 5. Parameters that were not explicitly set in the training script were left at the default values of the Stable-Baselines3 implementation [31].

The same observation vector, action space, and evaluation metrics were used when comparing PPO with A2C. This ensures that the comparison reflects differences between the optimization algorithms rather than differences in the underlying control task.

### 3.7. Evaluation Metrics

The evaluation uses metrics for formation accuracy, safety, dynamic synchronization, and actuator regularity. The basic formation-tracking error is as follows:

(53) $$e_{\mathrm{form}}\left(t\right)=d_{\mathrm{behind}}\left(t\right)={∥p_{F}\left(t\right)-p_{\mathrm{ref}}\left(t\right)∥}_{2}.$$

where the following hold:

· $e_{\mathrm{form}}\left(t\right)$—instantaneous formation-tracking error;
· $d_{\mathrm{behind}}\left(t\right)$—distance between the follower and the reference point behind the leader;
· $p_{F}\left(t\right)$—follower position;
· $p_{\mathrm{ref}}\left(t\right)$—desired reference position behind the leader;
· ${∥\cdot ∥}_{2}$—Euclidean norm.

For an episode of length *T*, the mean and maximum formation errors are computed as

(54) $${\bar{e}}_{\mathrm{form}}=\frac{1}{T}\sum_{t=1}^{T}e_{\mathrm{form}}\left(t\right),\;e_{\mathrm{form}}^{\max}=\max_{t}e_{\mathrm{form}}\left(t\right).$$

where the following hold:

· ${\bar{e}}_{\mathrm{form}}$—mean formation error over an episode;
· $e_{\mathrm{form}}^{\max}$—maximum formation error over an episode;
· *T*—episode length;
· $e_{\mathrm{form}}\left(t\right)$—instantaneous formation-tracking error.

Safety is evaluated using the direct leader–follower distance $d_{L}\left(t\right)$. The safety-violation rate is defined as

(55) $$V_{\mathrm{safe}}=\frac{1}{T}\sum_{t=1}^{T}I\left(d_{L}\left(t\right)<d_{\mathrm{safe}}\right),$$

where the following hold:

· $V_{\mathrm{safe}}$—safety-violation rate;
· *T*—episode length;
· I(·)—indicator function;
· $d_{L}\left(t\right)$—direct leader–follower distance;
· $d_{\mathrm{safe}}$—minimum admissible leader–follower distance.

In tables, this quantity is reported as a percentage. The cumulative severity of intrusions into the unsafe zone is as follows:

(56) $$S_{\mathrm{unsafe}}=\sum_{t=1}^{T}\max\left(0,d_{\mathrm{safe}}-d_{L}\left(t\right)\right).$$

where the following hold:

· $S_{\mathrm{unsafe}}$—cumulative safety-violation severity,
· *T*—episode length,
· $d_{\mathrm{safe}}$—minimum admissible leader–follower distance,
· $d_{L}\left(t\right)$—direct leader–follower distance.

Velocity synchronization is measured using the absolute difference between the follower forward velocity and the leader speed:

(57) $$E_{v}=\frac{1}{T}\sum_{t=1}^{T}\left|v_{F}\left(t\right)-v_{L}\left(t\right)\right|.$$

where the following hold:

· $E_{v}$—mean absolute velocity-tracking error;
· *T*—episode length;
· $v_{F}\left(t\right)$—follower forward velocity;
· $v_{L}\left(t\right)$—leader speed.

Control smoothness is evaluated at the actuator-command level using the change in normalized actions between consecutive time steps:

(58) $$S_{u}=\frac{1}{T-1}\sum_{t=2}^{T}{∥a_{t}-a_{t-1}∥}_{2}.$$

where the following hold:

· $S_{u}$—control-smoothness metric;
· *T*—episode length;
· $a_{t}$—normalized action vector at time step *t*;
· $a_{t-1}$—normalized action vector at the previous time step;
· ${∥\cdot ∥}_{2}$—Euclidean norm.

Here, lower $E_{v}$ and $S_{u}$ indicate better velocity matching and smoother wheel-command generation, respectively.

For additional analysis of abrupt leader maneuvers, the recovery time after a sudden change of leader direction can be defined as

(59) $$T_{\mathrm{rec}}=\min\left\{t:e_{\mathrm{form}}\left(t\right)<e_{\mathrm{thr}}\;\mathrm{for}\;N\;\mathrm{consecutive}\;\mathrm{steps}\right\}.$$

where the following hold:

· $T_{\mathrm{rec}}$—recovery time after a sudden leader-direction change;
· $e_{\mathrm{form}}\left(t\right)$—instantaneous formation-tracking error;
· $e_{\mathrm{thr}}$—error threshold defining recovered formation tracking;
· *N*—number of consecutive steps required below the threshold.

This metric is used in the generalization scenario to assess recovery after sharp turns and zigzag-like motion. Overall, the metrics separate tracking, safety, synchronization, and actuator regularity instead of relying only on cumulative reward.

## 4. Experiments and Results

This section presents the experimental evaluation of the proposed reinforcement-learning-based leader–follower controller. In contrast to the preliminary evaluation, which focused mainly on training curves and selected trajectory visualizations, the revised protocol provides a broader and more systematic assessment of the learned policies. The experiments include nominal testing, reward-weight sensitivity analysis, comparison with a classical distance–bearing PID baseline, robustness evaluation under observation and actuation disturbances, observation-space ablation, and generalization testing on an unseen maneuver sequence referred to as Scenario 17.

The evaluation was designed to answer four main questions. First, the reward-weight sensitivity analysis investigates how individual reward components affect safety, formation accuracy, velocity synchronization, and control smoothness. Second, the nominal comparison evaluates PPO against A2C and the classical PID baseline under the same observation and action spaces. Third, the robustness tests determine whether the trained policy remains reliable when observation noise, action delay, and wheel-slip-like actuation uncertainty are introduced at test time. Finally, Scenario 17 evaluates whether the learned controller can generalize to a trajectory containing abrupt leader maneuvers that differ from the nominal training paths.

The results are reported using task-level metrics rather than cumulative reward alone. The main evaluation criteria include collision occurrence, safety-violation rate, safety-violation severity, minimum leader–follower distance, mean and maximum formation error, velocity-tracking error, control smoothness, control jerk, recovery time, and success rate. Unless stated otherwise, the reported values are aggregated over multiple evaluation episodes and presented as mean values with standard deviations.

### 4.1. Experimental Protocol

The experimental evaluation used a separate test pipeline built around the trained policies and the MuJoCo leader–follower environment. Four test suites were considered: nominal evaluation on trajectories 0–15, robustness tests with disturbances introduced only at evaluation time, observation-space ablation, and Scenario 17 as an unseen composite trajectory.

In the nominal evaluation, each controller was tested on the standard trajectory set using 30 episodes per condition and a maximum episode length of 1500 steps. Scenario 17 uses a 6000-step horizon and a nominal leader speed of 1.0m/s. PPO, A2C, and the distance–bearing PID baseline all use the same compact observation vector and normalized two-dimensional action space.

Unless stated otherwise, reported standard deviations quantify episode-level variability, including differences between trajectories and initial conditions. They should not be interpreted as variability across independently trained random seeds. Robustness disturbances were introduced only during testing and include Gaussian observation noise, action delay, wheel-slip-like action scaling, and their combined form. The experimental test suites are summarized in Table 6.

The ablation study masks selected observation entries while keeping the trained policy unchanged. The tested variants remove leader speed, leader direction, follower velocity, or retain only distance and bearing. Scenario 17 then evaluates generalization on six maneuver segments, including a sharp turn and zigzag motion, which require rapid correction of bearing and formation distance. The following subsections report reward-weight sensitivity, baseline comparisons, robustness, ablation, and Scenario 17 results.

### 4.2. Reward-Weight Sensitivity Analysis

The first experiment evaluates how reward-weight selection affects learning and final leader–follower behavior. Seven PPO variants, R1–R7, modify the weights of formation tracking, safety-distance preservation, control-effort regularization, and bearing-angle shaping, while all other experimental settings remain unchanged. R1 is the baseline, R2–R3 emphasize safety, R4 emphasizes tracking, R5–R6 modify control-effort regularization, and R7 strengthens bearing-angle shaping.

**Figure 2:**
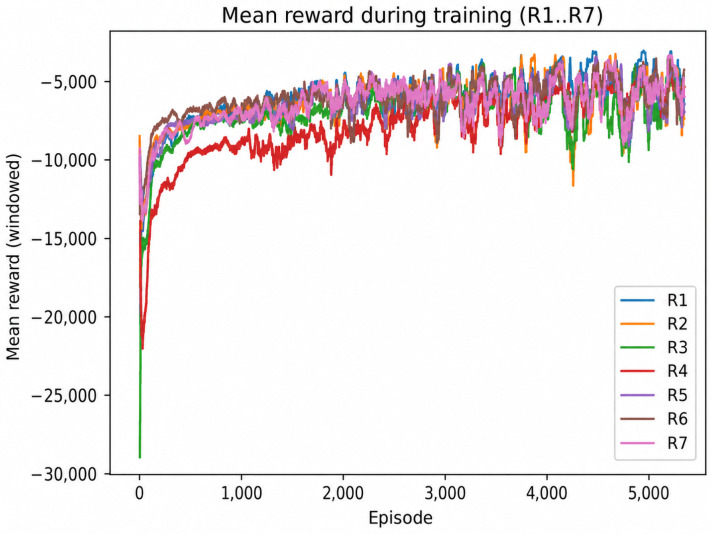
Mean episode reward during PPO training for reward variants R1–R7. Higher values, i.e., values closer to zero, indicate better policy performance because the reward function is composed primarily of penalty terms.

Figure 2 shows the smoothed mean episode reward during training. Since the reward is dominated by penalty terms, values closer to zero indicate better optimization. All variants improve rapidly at the beginning of training and then exhibit variant-specific convergence behavior.

The stability of the learning process was additionally assessed using the standard deviation of the reward signal. Let $R_{i}$ denote the reward obtained in episode *i*, and let $\bar{R}$ be the mean reward over *N* episodes. The reward variability is defined as

(60) $$\sigma_{R}=\sqrt{\frac{1}{N}\sum_{i=1}^{N}{\left(R_{i}-\bar{R}\right)}^{2}}.$$

where the following hold:

· $\sigma_{R}$—standard deviation of the reward signal;
· $R_{i}$—reward obtained in episode *i*;
· $\bar{R}$—mean reward over the analyzed set of episodes;
· *N*—number of episodes used to compute the statistic.

**Figure 3:**
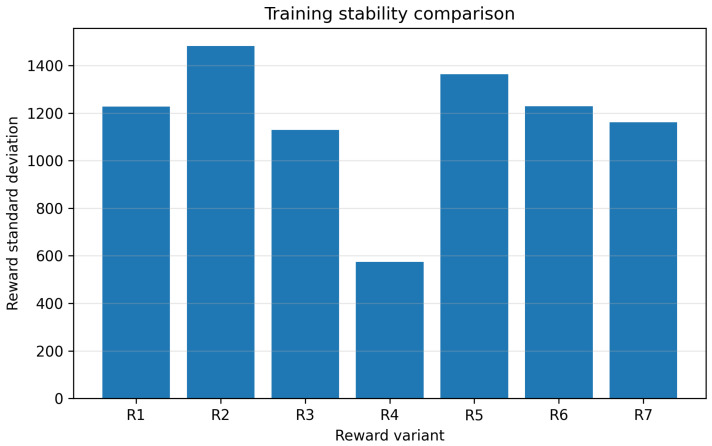
Reward variability during PPO training for reward variants R1–R7. Lower values indicate smaller fluctuations in the reward signal and therefore more stable learning dynamics.

Lower $\sigma_{R}$ indicates smaller reward oscillations. Figure 3 shows that R4 has the lowest reward variability, but this alone does not imply the best task behavior, because safety and formation accuracy must also be considered.

Table 7 summarizes task-level results over 10 evaluation episodes per variant. Because the reward is a weighted sum of penalties, less negative values indicate better optimization within a given formulation. However, raw reward values are not directly comparable across all variants and must be interpreted together with safety, tracking, synchronization, and smoothness metrics.

R6 achieved the highest final reward (−3351), the lowest mean formation error (0.331m), and the lowest velocity-tracking error (0.087m/s). However, it did not provide the lowest safety-violation rate. From the safety perspective, R2 and R3 both reached a safety-violation rate of 0.50% and avoided collisions, with R3 offering lower tracking and velocity errors than R2.

**Figure 4:**
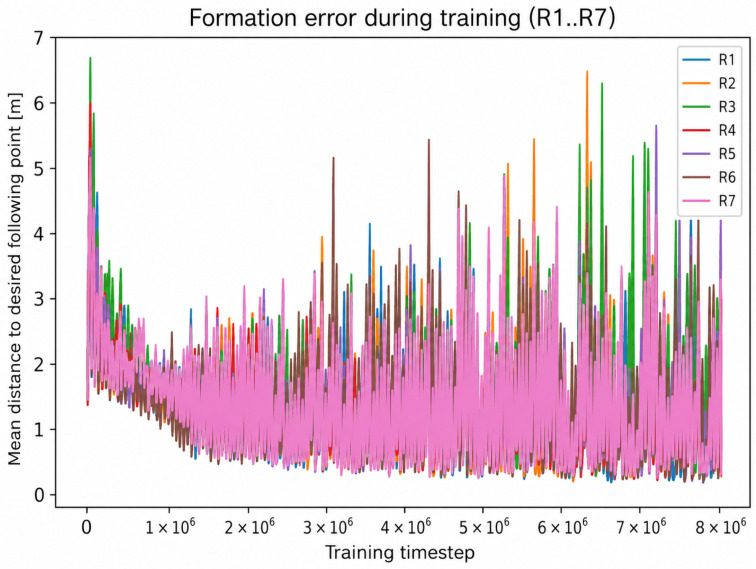
Mean distance to the desired following point during PPO training for reward variants R1–R7. Lower values indicate more accurate formation tracking.

R4 illustrates the limitation of using reward stability or control smoothness as the sole criterion for selecting a reward configuration. Although R4 produced the smoothest control signal, it also resulted in the highest safety-violation rate and collision count. Overall, R6 is preferable when average formation accuracy is dominant, whereas R3 provides the more conservative safety-oriented compromise. The formation-error evolution in Figure 4 supports this interpretation by showing intermittent error peaks during difficult trajectory fragments.

**Figure 5:**
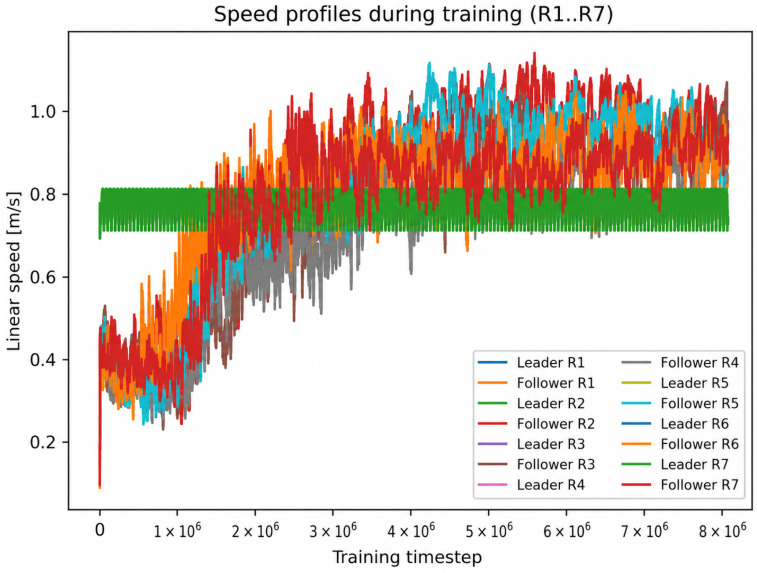
Leader and follower speed profiles during PPO training for reward variants R1–R7. The follower speed gradually approaches the leader-speed range, indicating improved dynamic synchronization during training.

Figure 5 shows that the follower speed gradually approaches the leader-speed range during training, indicating improved dynamic synchronization in addition to geometric tracking.

In summary, reward design strongly affects the safety–accuracy–regularity trade-off. R3 is the most conservative and balanced configuration, whereas R6 is preferable when minimizing average formation error is the dominant objective.

### 4.3. Nominal Comparison with PPO, A2C, and PID Baseline

The nominal comparison evaluates PPO, A2C, and the distance–bearing PID controller under the standard leader trajectories. Based on the reward-weight sensitivity analysis, R3 was selected as the safety-oriented PPO/A2C configuration and R6 as the accuracy-oriented configuration. All controllers were evaluated using the same observation vector, action space, trajectory set, actuator limits, and task metrics.

The comparison was performed over 480 nominal evaluation episodes for each controller. The reported metrics include collision rate, safety-violation rate, cumulative safety-violation severity, mean formation error, velocity-tracking error, and success rate. An episode is considered successful only if no collision occurs, the safety-violation rate remains below 1%, and the mean formation error remains below 0.75m; therefore, success rate is a strict task-level measure rather than a reward-based score.

Table 8 shows that the PPO controllers provide the most balanced task-level performance under nominal conditions. PPO_R6 obtains the lowest mean formation error, 1.353m, and the lowest velocity-tracking error, 0.274m/s. PPO_R3 gives slightly weaker tracking accuracy, but lower cumulative safety-violation severity than PPO_R6. The A2C variants reach comparable mean formation errors, but their collision and safety-violation metrics are much worse, leading to zero success rate. The PID baseline remains conservative with respect to safety-violation severity, but this comes at the cost of substantially larger formation error.

**Figure 6:**
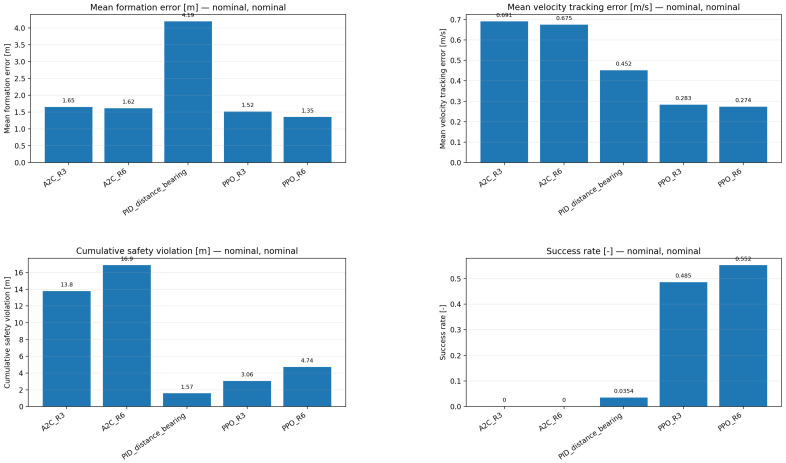
Nominal evaluation of A2C, PPO, and the distance–bearing PID baseline. The bar plots summarize formation accuracy, velocity synchronization, cumulative safety-violation severity, and success rate.

Figure 6 summarizes the same trend visually: PPO_R6 is the most accurate, PPO_R3 is the more conservative PPO variant, A2C fails the strict success criterion, and PID reduces violation severity but does not maintain the desired formation reliably.

**Figure 7:**
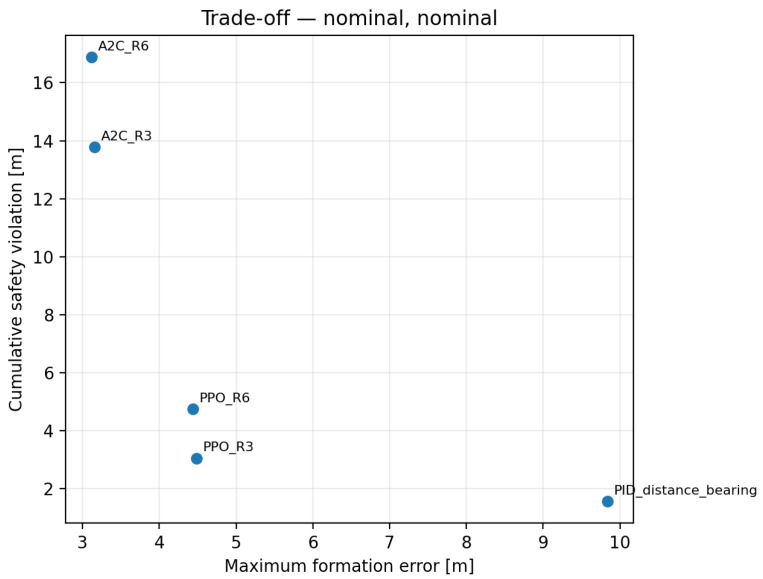
Trade-off between maximum formation error and cumulative safety-violation severity under nominal conditions. The preferred region is the lower-left part of the plot, where both tracking error and safety violation are low.

The trade-off plot in Figure 7 illustrates the multi-objective nature of the task. The PID baseline occupies a low-violation but high-error region, the A2C policies occupy a high-violation region, and the PPO variants remain closer to the desirable compromise region. Thus, PPO_R3 is preferable when safety-related criteria are prioritized, whereas PPO_R6 is preferable when formation accuracy and dynamic synchronization dominate.

### 4.4. Observation-Space Ablation Study

The observation-space ablation study evaluates how individual components of the compact six-dimensional observation vector affect the trained PPO policies. Selected observation entries were set to zero only during evaluation, while the environment state and task-level metrics were computed from the unmodified simulation state.

The full observation vector is defined as

(61) $$o_{t}={\left[\alpha_{t},{\bar{d}}_{L,t},{\bar{\omega }}_{F,t},{\bar{v}}_{F,t},{\bar{v}}_{L,t},\Delta \psi_{t}\right]}^{⊤}.$$

The Full 6D condition uses all components. The ablated variants remove leader speed ${\bar{v}}_{L,t}$, leader direction $\Delta \psi_{t}$, follower-velocity components ${\bar{v}}_{F,t}$ and ${\bar{\omega }}_{F,t}$, or retain only relative distance and bearing. PPO_R3 and PPO_R6 were selected as the safety-oriented and accuracy-oriented policies; A2C is not emphasized because it remained non-competitive in the nominal comparison.

Table 9 shows that the full 6D observation is the most reliable condition. PPO_R6 achieves the highest success rate and lowest mean formation error, whereas PPO_R3 retains lower cumulative safety-violation severity. The leader-direction component $\Delta \psi_{t}$ is the most safety-critical channel: masking it increases the safety-violation rate to 44.242% for PPO_R3 and 29.399% for PPO_R6.

**Figure 8:**
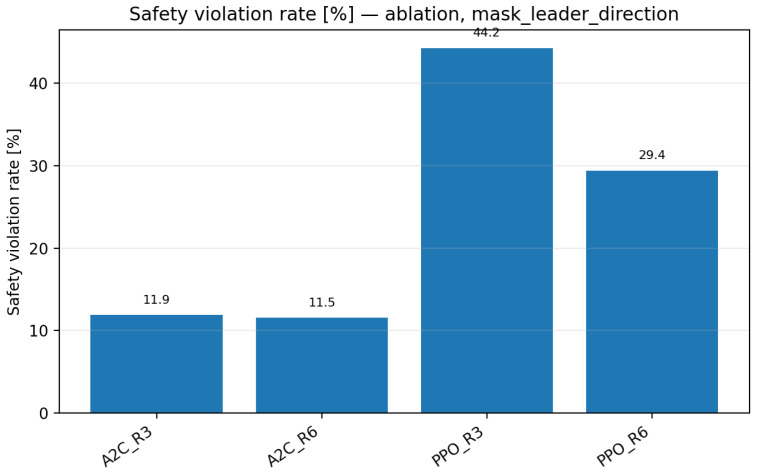
Effect of masking the leader-direction component on the safety-violation rate. Removing $\Delta \psi_{t}$ substantially increases safety violations, which indicates that the relative direction of leader motion is critical for maintaining a safe leader–follower configuration.

**Figure 9:**
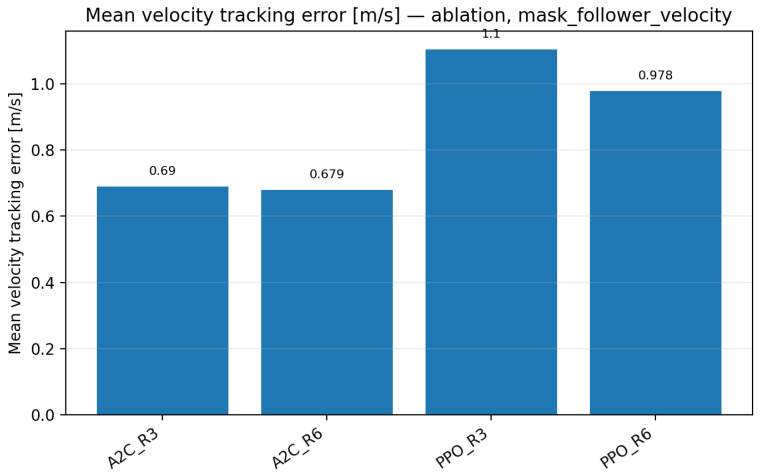
Effect of masking follower-velocity components on velocity-tracking error. Removing proprioceptive velocity information increases the mismatch between follower and leader motion, confirming its importance for dynamic synchronization.

**Figure 10:**
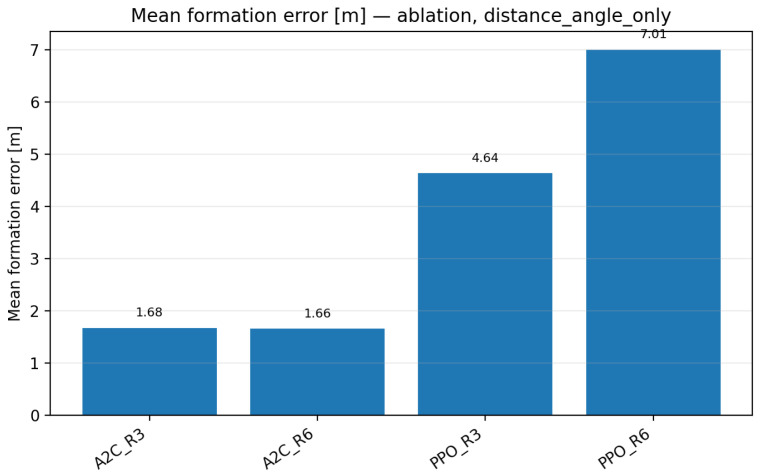
Formation error under the distance–angle-only observation condition. Retaining only relative distance and bearing is insufficient for reliable formation tracking, especially for the PPO policies that rely on velocity and leader-direction information.

Removing follower velocity mainly degrades formation accuracy and synchronization. The mean formation error increases to 5.129m for PPO_R3 and 4.529m for PPO_R6, confirming the importance of proprioceptive velocity feedback. The main ablation effects are visualized in Figure 8, Figure 9 and Figure 10.

Leader speed has a weaker effect on mean formation error, but it contributes to the combined success criterion. The distance–angle-only condition confirms that relative bearing and distance alone are insufficient for this dynamic task, as both PPO variants obtain zero success rate.

Overall, the compact observation vector is low-dimensional but not arbitrary: leader direction supports safety, follower velocity supports synchronization and accuracy, and leader speed helps satisfy the combined success criterion.

### 4.5. Robustness Under Observation and Actuation Disturbances

The robustness evaluation examines PPO_R3 and PPO_R6 under relaxed observation and actuation assumptions. The tests are motivated by sim-to-real and domain-randomization studies, where observation uncertainty, dynamics mismatch, and actuator-effectiveness changes commonly degrade performance [26,27,28]. Disturbances were introduced only during evaluation, without retraining or fine-tuning, and the distance–bearing PID controller was retained as a classical reference.

The suite includes one nominal reference and seven disturbed conditions: low/high Gaussian observation noise, one-/three-step action delay, mild/strong wheel-slip-like action scaling, and a combined uncertainty condition. Each controller was evaluated over 480 episodes per condition. The disturbances introduced during robustness evaluation can be formalized as follows. Observation noise is modeled as an additive Gaussian perturbation applied to the compact observation vector:

(62) $${\tilde{o}}_{t}=o_{t}+\epsilon_{t},\;\epsilon_{t}∼N\left(0,\sigma_{o}^{2}I\right).$$

where the following hold:

· ${\tilde{o}}_{t}$—disturbed observation vector used during evaluation;
· $o_{t}$—nominal observation vector returned by the environment;
· $\epsilon_{t}$—Gaussian observation-noise vector;
· $\sigma_{o}$—standard deviation of the observation noise;
· *I*—identity matrix matching the observation dimension.

Actuation delay is modeled by executing an action selected in a previous control step:

(63) $$a_{t}^{\mathrm{exec}}=a_{t-k}.$$

where the following hold:

· $a_{t}^{\mathrm{exec}}$—action actually executed by the simulator at time step *t*;
· $a_{t-k}$—policy action generated *k* control steps earlier;
· *k*—number of delayed control steps.

Wheel-slip-like actuation uncertainty is approximated by random scaling of the executed action:

(64) $$a_{t}^{\mathrm{exec}}=\lambda_{t}a_{t},\;\lambda_{t}∼U\left(\lambda_{\min},\lambda_{\max}\right).$$

where the following hold:

· $a_{t}^{\mathrm{exec}}$—scaled action executed by the simulator;
· $a_{t}$—nominal action generated by the policy;
· $\lambda_{t}$—random action-scaling coefficient;
· $U(\lambda_{\min},\lambda_{\max})$—uniform distribution over the scaling interval.

Table 10 reports the main robustness results for PPO_R3 and PPO_R6. To keep the table readable, three representative task-level metrics are shown: cumulative safety-violation severity, mean formation error, and success rate. These metrics summarize the main safety–accuracy trade-off under non-nominal observation and actuation conditions. Lower values are preferable for safety-violation severity and formation error, whereas higher values are preferable for success rate. Additional metrics, including collision rate, safety-violation rate, velocity-tracking error, control smoothness, and control jerk, were also computed and used in the interpretation.

Table 10 indicates that both PPO variants retain comparable aggregate quality under the tested disturbances, but the large standard deviations show that performance remains trajectory-dependent. The results should therefore be interpreted as an aggregate comparison over the evaluated trajectory set, not as evidence of uniform robustness in every episode.

PPO_R3 generally maintains lower cumulative safety-violation severity than PPO_R6; under combined uncertainty, the values are 1.546m and 4.240m, respectively. PPO_R6, in turn, often preserves lower mean formation error, including 1.182m under strong wheel-slip-like scaling and 1.221m under combined uncertainty. Thus, the same safety–accuracy trade-off observed in nominal testing remains visible under disturbances.

Action delay and observation noise expose different weaknesses. A three-step delay increases the PPO_R6 mean formation error to 1.660m, while high observation noise increases control-smoothness values to 0.166 for PPO_R3 and 0.194 for PPO_R6. The PID baseline remains comparatively conservative but inaccurate, with mean formation error of approximately 4.15–4.75m and near-zero success. Overall, the robustness tests support cautious conclusions: the PPO policies do not collapse under the evaluated disturbances, but no direct sim-to-real claim can be made without physical robot validation.

### 4.6. Generalization Test Scenario

In order to evaluate the generalization capability of the trained policies, an independent testing scenario was designed. The purpose of this experiment is to verify whether the follower robot is able to maintain the desired formation when the leader executes a trajectory that differs from those used during the training phase.

The scenario consists of a sequence of motion primitives forming a closed trajectory executed by the leader robot. During the test the leader moves with a constant forward velocity and performs a series of maneuvers combining curvilinear motion, straight-line segments, and oscillatory movement patterns. Throughout the experiment the follower robot is required to continuously adapt its motion in order to maintain the desired relative position behind the leader.

**Figure 11:**
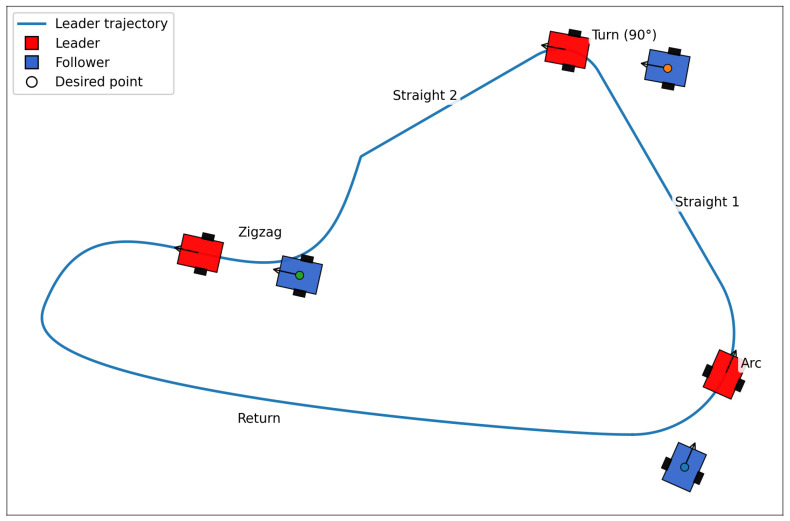
Leader trajectory used in the generalization experiment (Scenario 17).

As illustrated in Figure 11, the trajectory of the leader is composed of several motion primitives designed to introduce different types of motion patterns and dynamic conditions for the follower robot. The trajectory consists of several motion primitives listed below.

1. Circular arc —the leader follows a fragment of a circular trajectory corresponding to approximately $120^{\circ }$ of rotation. This segment evaluates the ability of the follower to track smooth curvilinear motion while maintaining the desired formation geometry.
2. Straight segment—the leader moves along a straight-line trajectory with constant velocity, allowing the follower robot to stabilize its position relative to the desired reference point in the formation.
3. Sharp turn—the leader performs a turn of approximately $90^{\circ }$ along a short circular arc with a smaller radius. This maneuver introduces a rapid change of heading that requires the follower to react promptly in order to maintain the desired relative configuration.
4. Second straight segment—another straight motion segment is executed after the turning maneuver. This part of the trajectory is used to evaluate the ability of the follower to regain a stable formation after a sudden change in the leader’s heading.
5. Zigzag motion—the leader moves forward while simultaneously performing lateral oscillations described by a sinusoidal function. This segment introduces periodic lateral disturbances and provides a more challenging tracking task for the follower robot.
6. Return segment—finally, the trajectory smoothly returns to the initial position using a fifth–order polynomial interpolation that ensures continuity of both position and velocity profiles.

The complete trajectory forms a closed motion cycle, which allows the experiment to be repeated for multiple episodes without introducing discontinuities in the leader motion. Such structure ensures consistent testing conditions across different trained models.

During each evaluation episode the follower robot starts from a randomized initial pose. This procedure prevents the learned policies from overfitting to a specific initial configuration and enables a more reliable assessment of their generalization capability.

The performance of the trained models is evaluated using the average distance between the follower robot and the desired reference point located behind the leader, as well as the distance between the follower and the leader itself. These metrics provide quantitative indicators of both formation accuracy and operational safety during the execution of the trajectory.

### 4.7. Comparison of PPO and A2C

An auxiliary experiment compared proximal policy optimization (PPO) with advantage actor–critic (A2C) in the considered leader–follower task. The goal was not to provide an exhaustive reinforcement-learning benchmark, but to verify whether the choice of PPO as the primary optimization algorithm is justified. Both algorithms were trained and evaluated using the same environment, observation and action spaces, and baseline reward variant R1.

Both methods belong to the actor–critic family and can handle continuous-control problems. PPO differs mainly through its clipped policy-update mechanism, which is intended to limit excessively large policy changes. This property is relevant here because unstable policy updates may cause oscillatory motion, degraded formation tracking, or more frequent safety-zone violations.

**Figure 12:**
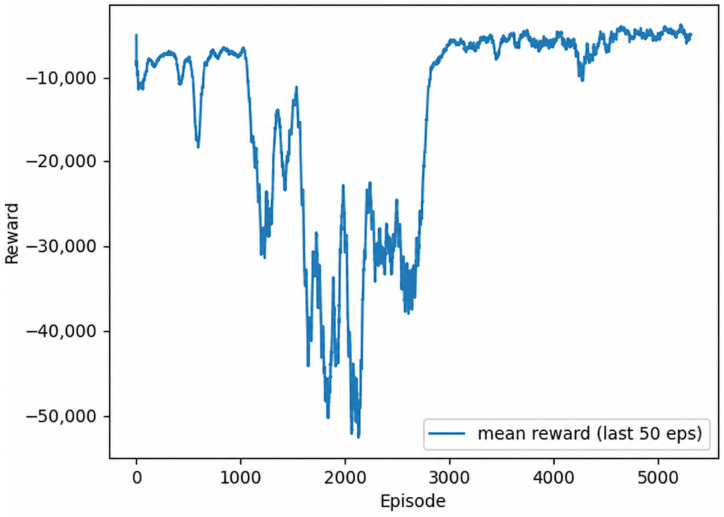
Mean episode reward during A2C training for the baseline reward variant R1. The plotted value represents the moving average over the last 50 episodes.

**Figure 13:**
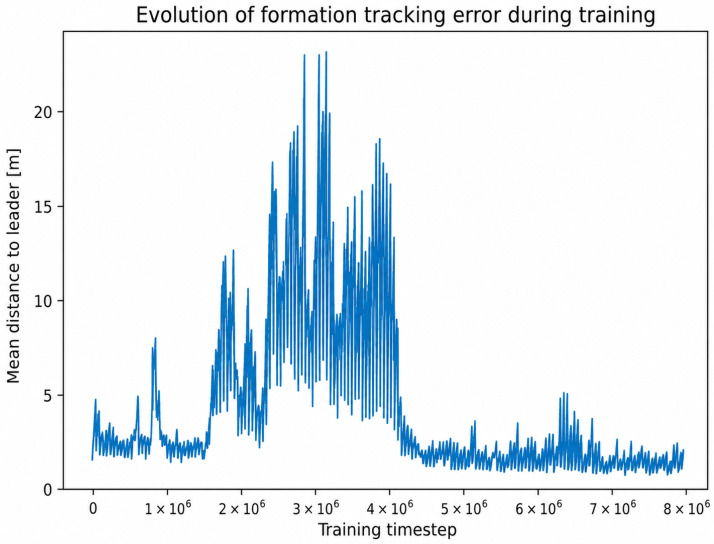
Evolution of formation tracking error during A2C training for the baseline reward variant R1.

**Figure 14:**
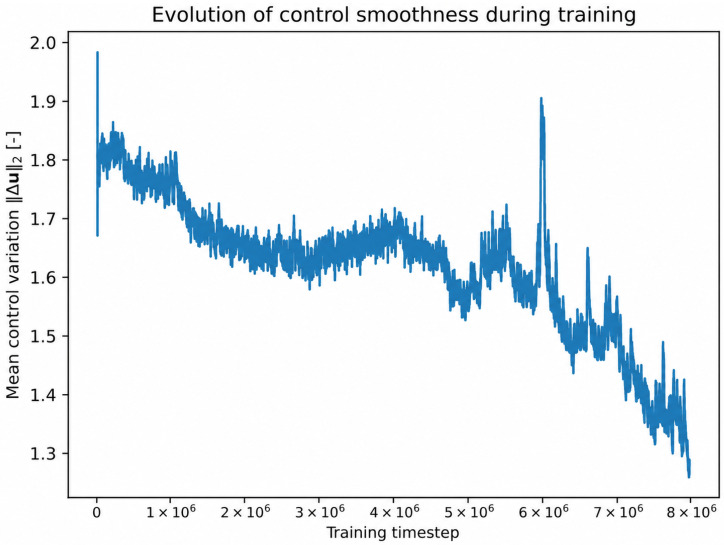
Evolution of control smoothness during A2C training for the baseline reward variant R1. Lower values correspond to smoother control signals.

**Figure 15:**
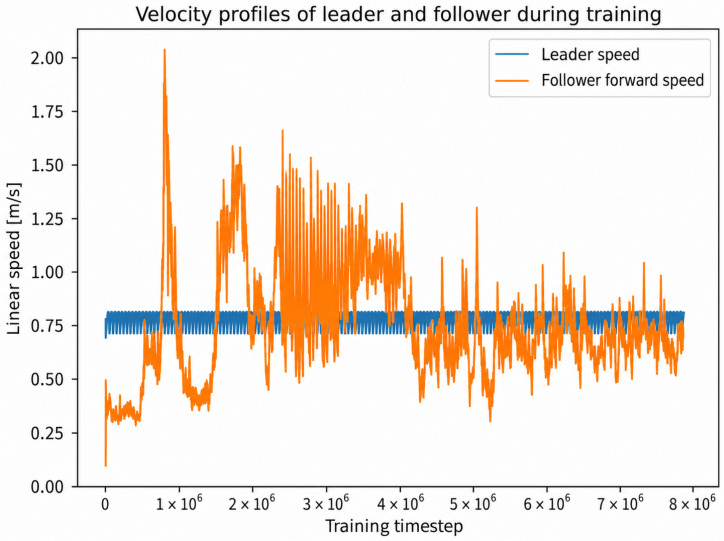
Velocity profiles of the leader and follower during A2C training for the baseline reward variant R1.

**Figure 16:**
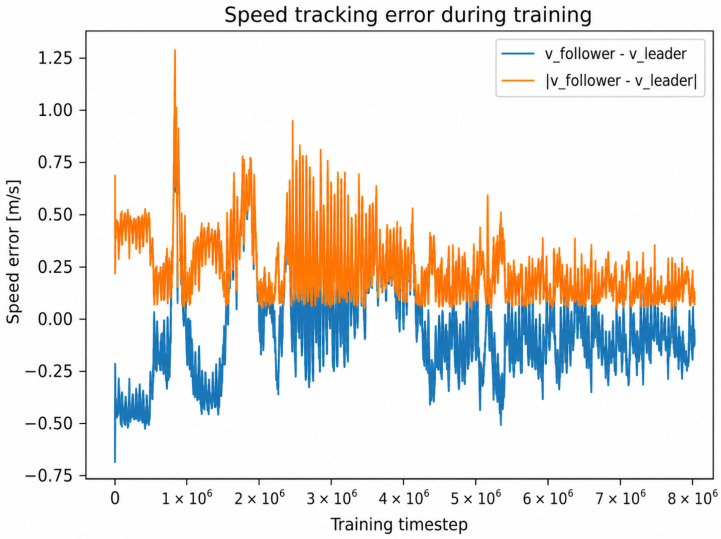
Speed tracking error during A2C training for the baseline reward variant R1. The plot shows the discrepancy between follower and leader velocity over the course of training.

The comparison used the same task-oriented indicators as the reward-weight sensitivity analysis, including collision count, safety violation rate, mean formation error, velocity-tracking error, control smoothness, and reward variability. The A2C training curves are shown in Figure 12, Figure 13, Figure 14, Figure 15 and Figure 16.

The reward curve indicates substantial variability during the intermediate phase of A2C training, followed by recovery and gradual stabilization at a higher reward level. This suggests that A2C can discover a useful control strategy, but its convergence path is less regular.

Figure 13 shows that the formation error decreases in the final training phase, although pronounced intermediate oscillations remain visible. This behavior is undesirable in a safety-sensitive control task, where stable learning dynamics are important.

The control-smoothness metric improves over training, but local fluctuations indicate that abrupt wheel-command changes are still present in parts of the learning process.

The velocity plots show gradual improvement in follower–leader synchronization, but also confirm substantial fluctuations before the final training phase. Overall, A2C improves reward, formation maintenance, smoothness, and velocity matching over time, yet the learning process is noticeably oscillatory. PPO was therefore adopted in the main experiments because its clipped update provides a more conservative optimization mechanism, which is better aligned with the safety-sensitive and trajectory-changing nature of the task. A2C remains a meaningful baseline, but the comparison supports PPO as the primary algorithm used in the study.

## 5. Conclusions

This paper addressed leader–follower navigation for a wheeled mobile robot under dynamic leader maneuvers. A PPO-based follower was trained in a MuJoCo environment to maintain a desired position behind the leader while penalizing unsafe proximity.

The main contribution is a modular reward formulation combining formation tracking, collision avoidance, control-effort regularization, and bearing-angle shaping. The reward-weight study showed that no variant dominates all criteria: R3 provides a more conservative safety-oriented trade-off, whereas R6 improves average formation accuracy and velocity synchronization.

In the simulated tests, PPO achieved a more favorable safety–tracking trade-off than the considered A2C variants and the classical distance–bearing PID baseline. However, collision-free behavior was not guaranteed in all episodes, so the results should be interpreted as empirical simulation evidence rather than proof of deployment-ready safety.

The ablation and robustness studies indicate that the learned behavior depends strongly on the compact observation structure, especially the relative direction of leader motion, and that test-time disturbances produce trajectory-dependent variability. Future work will address multi-robot formations, sample efficiency, across-seed statistical validation, and sim-to-real transfer through systematic domain randomization and real-robot experiments [26,27,28].

## Figures and Tables

**Table 1:** Geometric and physical parameters of the simulated leader–follower environment.

Object	MuJoCo Type	Dimensions/Parameters	Mass
Follower chassis	box	0.40×0.30×0.10m	–
Drive wheel (×2)	cylinder	radius $r_{w}=0.07\;m$, width 0.04m	1.5kg
Lateral wheel spacing	–	L≈0.346m	–
Passive support wheel	sphere	radius 0.02m	0.01kg
Leader body	kinematic mocap object	visual body with wheel-like markers	–
Formation reference point	mocap sphere	radius 0.05m	–
Wheel actuator range	motor	$u_{L},u_{R}\in \left[-10,10\right]$	–

**Table 2:** Random-seed training stability for shortened PPO_R3 training runs with seeds s∈{0,1,2}. Reward values are negative because the objective is dominated by penalty terms; therefore, less negative values indicate better training performance.

Seed	Initial Reward	Final Reward	Improvement [%]	Final Error [m]	Velocity-Tracking Error [m/s]	Smoothness
0	−11,335.6	−6065.4±159.2	46.5	1.727	1.315	1.668
1	−10,901.4	−6876.6±562.2	36.9	1.728	1.269	1.620
2	−15,026.8	−7545.2±504.4	49.8	2.003	1.383	1.600
Mean ± std	−12,421.2 ± 2266.9	−6829.1±741.0	44.4±6.7	1.819±0.159	1.322±0.058	1.630±0.035

**Table 3:** Neural-network input, output, and policy representation.

Element	Configuration
Policy type	Feed-forward actor–critic multilayer perceptron
Implementation	Stable-Baselines3 MlpPolicy
Input dimension	6
Output dimension	2
Actor hidden layers	[64,64] fully connected layers
Critic hidden layers	[64,64] fully connected layers
Activation function	Hyperbolic tangent, Tanh
Actor output	Parameters of a diagonal Gaussian wheel-command distribution
Critic output	Scalar value estimate $V_{\phi }\left(o_{t}\right)$
Optimizer	Adam
Recurrent layers	Not used
Image encoder	Not used
Action range	Normalized ${\left[-1,1\right]}^{2}$

**Table 4:** Reward-function variants used in the sensitivity study.

Variant	$w_{f}$	$w_{c}$	$w_{a}$	$w_{\theta }$	Interpretation
R1	2.0	12.0	0.30	0.01	Baseline configuration
R2	2.0	16.0	0.30	0.01	Increased safety penalty
R3	2.0	20.0	0.30	0.01	Strong safety penalty
R4	3.0	12.0	0.30	0.01	Stronger formation tracking
R5	2.0	12.0	0.60	0.01	Stronger control-effort regularization
R6	2.0	12.0	0.15	0.01	Weaker control-effort regularization
R7	2.0	12.0	0.30	0.03	Stronger bearing-angle shaping

**Table 5:** PPO training configuration used in the experiments.

Parameter	Value
Algorithm	PPO
Policy	MlpPolicy
Number of parallel environments	16
Training path identifiers	0–15
Total time steps per reward variant	$3\times 10^{6}$
Rollout length, n_steps	1024 per environment
Rollout samples per update	16×1024= 16,384
Batch size	512
Number of optimization epochs, n_epochs	10
Learning rate	$3\times 10^{-4}$
Discount factor, γ	0.99
GAE parameter, λ	0.95
PPO clipping range	0.20
Entropy coefficient	0.0
Value-function coefficient	0.5
Maximum gradient norm	0.5
Advantage normalization	Enabled
Optimizer	Adam
Maximum episode length	1500 environment steps
Safety threshold	0.5m

**Table 6:** Overview of the experimental test suites used in the evaluation.

Test Suite	Objective	Output
Nominal	Standard leader trajectories.	Reward sensitivity, PPO/A2C/PID comparison.
Robustness	Test-time noise, delay, and wheel-slip-like scaling.	Safety, error, smoothness, and success under disturbances.
Ablation	Masked observation components.	Performance drop after removing leader speed, leader direction, follower velocity, or using distance–angle only.
Scenario 17	Unseen composite path with smooth and abrupt maneuvers.	Trajectory plots and segment-level formation, safety, and recovery metrics.

**Table 7:** Reward-weight sensitivity analysis for PPO variants R1–R7 under nominal evaluation conditions. Values are reported as mean ± standard deviation over 10 evaluation episodes.

Variant	Collision Count	Safety Violation Rate [%]	Mean Error [m]	Max Error [m]	Velocity Error [m/s]	Final Reward
R1	0.40±1.26	1.70±4.20	0.684±0.005	1.478±0.002	0.114±0.001	−5729
R2	0.00±0.00	0.50±1.10	0.612±0.005	1.278±0.001	0.114±0.001	−6708
R3	0.00±0.00	0.50±1.10	0.527±0.002	0.912±0.000	0.095±0.001	−4496
R4	0.50±1.27	12.30±37.30	0.564±0.004	1.150±0.018	0.116±0.002	−4551
R5	0.10±0.32	3.20±6.00	0.602±0.004	1.314±0.007	0.118±0.002	−6264
R6	0.00±0.00	3.30±8.90	0.331±0.005	1.254±0.006	0.087±0.002	−3351
R7	0.10±0.32	3.50±9.40	0.646±0.007	1.462±0.004	0.119±0.003	−5234

**Table 8:** Nominal comparison of PPO, A2C, and the distance–bearing PID baseline. Results are reported as mean ± standard deviation over 480 evaluation episodes. Lower values are preferable for all metrics except success rate.

Controller	Collision Rate [-]	Safety-Violation Rate [%]	Cumulative Safety-Violation Severity [m]	Mean Formation Error [m]	Velocity-Tracking Error [m/s]	Succes Rate [-]
A2C_R3	0.856±0.351	11.801±16.635	13.783±26.513	1.654±0.897	0.691±0.462	0.000±0.000
A2C_R6	0.883±0.321	14.137±19.189	16.875±29.640	1.616±0.848	0.675±0.465	0.000±0.000
PID	0.342±0.475	0.782±2.048	1.575±3.646	4.193±6.978	0.452±0.341	0.035±0.185
PPO_R3	0.456±0.499	3.508±10.240	3.056±8.700	1.518±4.168	0.283±0.183	0.485±0.500
PPO_R6	0.423±0.495	4.251±10.724	4.742±11.601	1.353±3.233	0.274±0.236	0.552±0.498

**Table 9:** Observation-space ablation results for PPO_R3 and PPO_R6. Results are reported as the mean ± standard deviation over 480 evaluation episodes per condition. Lower values are preferable for safety violation, severity, formation error, and velocity error, whereas higher values are preferable for success.

Policy	Observation Condition	Safety Viol. [%]	Severity [m]	Mean Error [m]	Velocity Error [m/s]	Success [-]
PPO_R3	Full 6D	3.508±10.240	3.056±8.700	1.518±4.168	0.283±0.183	0.485±0.500
PPO_R3	No leader speed	0.837±2.001	1.435±3.947	1.812±2.231	0.369±0.231	0.000±0.000
PPO_R3	No leader direction	44.242±36.854	62.355±57.285	4.479±6.955	0.426±0.238	0.000±0.000
PPO_R3	No follower velocity	4.279±11.177	4.956±10.226	5.129±5.570	1.104±0.534	0.046±0.209
PPO_R3	Distance–angle only	4.044±9.791	6.062±17.244	4.639±2.936	1.296±0.843	0.000±0.000
PPO_R6	Full 6D	4.251±10.724	4.742±11.601	1.353±3.233	0.274±0.236	0.552±0.498
PPO_R6	No leader speed	6.593±12.966	9.554±25.322	1.388±1.030	0.379±0.285	0.044±0.205
PPO_R6	No leader direction	29.399±30.130	28.785±43.494	2.714±4.205	0.388±0.213	0.000±0.000
PPO_R6	No follower velocity	4.118±9.596	3.675±9.413	4.529±5.209	0.978±0.570	0.006±0.079
PPO_R6	Distance–angle only	5.428±18.033	10.738±41.560	7.005±3.961	1.597±0.864	0.000±0.000

**Table 10:** Robustness evaluation of PPO_R3 and PPO_R6 under observation and actuation disturbances. Results are reported as mean ± standard deviation over 480 evaluation episodes per condition.

Cond.	R3 Cumulative Safety Violation Severity [m]	R3 Mean Formation Error [m]	R3 Succes Rate	R6 Cumulative Safety Violation Severity [m]	R6 Mean Formation Error [m]	R6 Succes Rate
Nominal	3.056±8.700	1.518±4.168	0.485±0.500	4.742±11.601	1.353±3.233	0.552±0.498
Noise low	2.838±8.278	1.436±3.955	0.487±0.500	4.698±11.441	1.484±3.523	0.556±0.497
Noise high	2.641±7.740	1.494±4.143	0.475±0.500	4.850±11.806	1.346±3.179	0.562±0.497
Delay 1	2.665±7.885	1.501±4.225	0.502±0.501	4.149±9.785	1.205±2.720	0.554±0.498
Delay 3	2.129±7.258	1.506±4.063	0.563±0.497	3.039±6.328	1.660±2.858	0.475±0.500
Slip mild	2.347±7.026	1.461±3.997	0.492±0.500	4.511±10.747	1.291±3.066	0.565±0.496
Slip strong	1.442±6.375	1.516±3.976	0.525±0.500	4.533±10.998	1.182±2.742	0.552±0.498
Combined	1.546±6.557	1.267±3.449	0.527±0.500	4.240±9.336	1.221±2.708	0.569±0.496

Table 10 reports the cumulative safety-violation severity, mean formation error, and success rate for PPO_R3 and PPO_R6. These metrics summarize the main safety–accuracy trade-off under non-nominal observation and actuation conditions.

## Data Availability

Data and code supporting the findings of this study are available from the corresponding author upon reasonable request.
